# Examination of the technological properties of newly isolated strains of the genus *Lactobacillus* and possibilities for their application in the composition of starters

**DOI:** 10.1080/13102818.2014.918701

**Published:** 2014-07-10

**Authors:** Rositsa Denkova, Svetla Ilieva, Zapryana Denkova, Ljubka Georgieva, Albert Krastanov

**Affiliations:** ^a^ Department of Biotechnology, Faculty of Biology, Sofia University ‘St. Kliment Ohridski’, Sofia, Bulgaria; ^b^ Department of Microbiology, Faculty of Technology, University of Food Technologies, Plovdiv, Bulgaria; ^c^ Institute of Cryobiology and Food Technology, Bulgarian Academy of Sciences, Sofia, Bulgaria

**Keywords:** *Lactobacillus*, sourdough, starter, bread, spoilage, food safety

## Abstract

The ability of four *Lactobacillus* strains – *Lactobacillus brevis* LBRZ7 (isolated from fermented cabbage), *Lactobacillus plantarum* LBRZ12 (isolated from fermented cabbage), *Lactobacillus fermentum* LBRH9 (of human origin) and *Lactobacillus casei* ssp. *rhamnosus* LBRC11 (isolated from home-made cheese) – to grow in flour/water environment and to accumulate high concentrations of viable cells was examined. Two starters for sourdough were created for lab-scale production of wheat bread: a two-strain starter and a four-strain starter. Wheat bread with improved properties – greater loaf volume, enhanced flavour and softer and brighter crumb – was obtained from the 7% four-strain starter sourdough. The addition of sourdough in the production of wheat bread affected positively the technological and organoleptic characteristics of the final bread by inhibiting the growth of wild yeasts and mold and *Bacillus* spores without the addition of preservatives. The inclusion of 15% of the four-strain starter sourdough in the bread-making process led to enhanced safety and longer shelf life of the baked bread.

## Introduction

A food can be regarded as functional if, beyond its inherent nutritional effects, it does satisfactorily demonstrate to affect beneficially one or more target functions in the body in a way that is relevant to either the state of well-being and health or to the reduction of the risk of a disease.[1,2] To increase their effect, mainly selected strains of lactic acid bacteria (LAB) are incorporated in the composition of functional foods to improve the taste, the aroma and the shelf life of the final products.

Bread is an important source of nutrients in human diet; especially of carbohydrates, fibre, proteins and some minerals (magnesium, phosphorus, iron).[3–5] The freshness of bread depends on its flavour and appearance, the crispness of the crust, the hardness of the crumb and the volume of the loaf; with its taste being considered the most important criterion for consumer acceptance.[6]

The shelf life of bakery products is very short.[7] Microbial spoilage of bakery products is a result of bacterial and fungal growth. The most common fungal species causing fungal spoilage belong to the genera *Aspergillus*, *Fusarium* and *Penicillium*.[8,9] Roping of bread caused by *Bacillus* sp., especially *Bacillus subtilis* and *Bacillus licheniformis*, can become noticeable within 12–24 h after the loaf is baked. This type of spoilage is initially noticed as an unpleasant odour, followed by a discoloured, sticky soft bread crumb caused by the breakdown of starch and proteins by microbial amylases and proteases, and by the production of extracellular, slimy polysaccharides.[10–12] When the cell counts (colony-forming units (cfu)) of *B. subtilis* and *B. licheniformis* reach over 10^5^ cfu/g, they present a potential risk of foodborne diseases.[13]

Among the various physical and chemical methods for preservation of baked goods from microbial spoilage, sourdough addition turns out to be the best preservation procedure, meeting the growing consumer demands for natural and additive-free foods.[13–16] Sourdough fermentation is also central to the flavour, as chemically acidified bread and breads prepared with pure commercial starter cultures do not score well in sensory preference assessments.[17–19]

The addition of starter sourdough brings about a wide range of improvements in the nutritional values [20,21] of the bread, the loaf specific volume and crumb structure,[7,22–24] the flavour and the shelf life.[7,24–29] The elongation of the shelf life is due to the prevention of microbial spoilage [23,30–32] as well as to the delay of the staling process.[33] These positive effects result from the metabolic activities of the micro-organisms in the composition of sourdough, including proteolysis, lactic acid fermentation, production of exopolysaccharides and synthesis of antimicrobial and volatile components.[7,13,34]

Mixed cultures of LAB and yeasts vary in composition in sourdough sponges. The use of mixed cultures has a number of important advantages, such as improved flavour and texture and freshness retained for longer compared to baker's yeast bread.[17,35] In such mixed cultures, yeasts act mainly as leavening agents, while LAB contribute mainly to the flavouring compounds of bread. Stable co-metabolism between LAB and yeasts is common in many foods, enabling the utilization of substrates that are otherwise non-fermentable (for example, starch) by individual micro-organisms and, thus, increasing the microbial adaptability to complex food ecosystems.[17,33,36,37]

The aim of the present study was to examine the technological characteristics of four *Lactobacillus* strains of different origin for their application in the composition of starters for sourdough for the production of preservative-free wheat bread with improved properties.

## Materials and methods

### Bacterial strains

Four strains of the genus *Lactobacillus* were used. The strains were isolated by us and are currently included in the collection of micro-organisms of the Department of Microbiology at the University of Food Technologies (Plovdiv, Bulgaria). The four strains were identified as *Lactobacillus brevis* LBRZ7 (isolated from fermented cabbage), *Lactobacillus plantarum* LBRZ12 (isolated from fermented cabbage), [38] *Lactobacillus fermentum* LBRH9 (of human origin) and *Lactobacillus casei* ssp. *rhamnosus* LBRC11 (isolated from home-made cheese).[39]

### Growth media

Standard growth media were used: De Man–Rogosa–Sharpe (MRS)-broth medium (Scharlau);[40] MRS-agar medium (MRS-broth medium + 2% agar (Scharlau)); LAPTg10-broth medium;[39] LAPTg10-agar medium;[39] LBG-agar medium.[39]

### Cultivation and storage conditions

All tested strains were isolated from a single colony and were grown in MRS-broth medium for 24 h in order to obtain pure cultures. For activation of the studied strains, they are cultured in liquid medium (MRS-broth) and on agar medium (MRS-agar) at 30 °C for *L. brevis* LBRZ7 and *L. plantarum* LBRZ12, and at 37 ºC for *L. casei* ssp. *rhamnosus* LBRC11 and *L. fermentum* LBRH9 for 18–24 h.

### Preparation of single-strain sourdough

MRS-broth 10 cm^3^ aliquots were inoculated with each *Lactobacillus* strain (1%) and incubated at 30 °C for *L. brevis* LBRZ7 and *L. plantarum* LBRZ12, or 37 °C for *L. casei* ssp. *rhamnosus* LBRC11 and *L. fermentum* LBRH9 for 24 h. Then, the biomass was collected by centrifugation (6000×g, 15 min, 4 °C) and the pellet was resuspended to the initial volume with sterile saline solution. The obtained cell suspension was used to inoculate the flour/water mixture. The changes in the concentration of viable LAB cells and in the titratable acidity of the sourdoughs were monitored daily by repeated kneading every 24 h over a period of 96 h of cultivation at 30 or 37 ºC, as follows: day 1: 44% flour to 56% tap water (40 ºC) and 10% cell suspension; day 2 to day 5: 25% sourdough from the previous day to 75% fresh flour/water mixture. A control sample without starter was prepared as well.

The LAB counts were determined by appropriate tenfold dilutions and plating on coloured LAPTg10-agar medium. A standard method [41] was used for measurement of the total titratable acidity (TTA).

### Preparation of sourdoughs with multi-strain starters

Two multi-strain starters were used. The two-strain combination contained *L. casei* ssp. *rhamnosus* LBRC11 and *L. brevis* LBRZ7 in a 3:7 ratio and the four-strain combination contained *L. plantarum* LBRZ12, *L. casei* ssp. *rhamnosus* LBRC11, *L. brevis* LBRZ7 and *L. fermentum* LBRH9 in a 2:1:1:1 ratio. To obtain sourdough with a multi-strain starter, 24-h suspensions of the strains were mixed and homogenized. Then, they were centrifuged and the cell pellet was resuspended with sterile saline solution to the initial volume of the mixed suspension. This cell suspension was used for inoculation of the flour/water mixture. The concentration of the inoculum of the ‘four-strain’ combination is 5.0 ×10^9^ cfu/cm^3^, while one of the inocula of the ‘two-strain’ combination is 9.5 × 10^8^ cfu/cm^3^. The accumulation of biomass and the change in the acidity of the sourdoughs during repeated kneading every 24 h over a period of 96 h is determined. At the 48th hour, 0.1% bakery yeast (*Saccharomyces cerevisiae*) is added to each of the two sourdoughs. The changes in the concentration of LAB viable cells, yeasts and molds, and in the titratable acidity of the two types of sourdough were monitored by repeated kneading every 24 h over a period of 96 h of cultivation at 30 ºC, as described above. At 48 h of repeated daily kneading, 0.1% yeast sponge (baker's yeast, *S. cerevisiae*) was added to each of the two multi-strain sourdoughs.

The viable cell counts were determined by appropriate tenfold dilutions and plating on coloured LAPTg10-agar medium for LAB or on LBG-agar for *S. cerevisiae*, ‘wild’ yeasts and molds. TTA was determined by a standard method.[41]

### Antimicrobial activity assay

The antimicrobial activity of sourdough against saprophytic micro-organisms was determined by the agar diffusion method. The two-strain and the four-strain starter sourdoughs were assayed. A 1:1 dilution of sourdough to saline solution was prepared. The antimicrobial activity of the sourdough was tested against the following saprophytic test micro-organisms: bacteria (*B. subtilis*), yeasts (*S. cerevisiae*) and molds (*Aspergillus niger*, *Penicillium* sp., *Rhizopus* sp.). A suspension of each of the test micro-organisms (10^6^–10^7^ cfu/cm^3^) was inoculated in Petri dishes with agar medium and after it solidified, agar wells (6 mm) were prepared. Then, 0.06 cm^3^ of the dilutions were pipetted in the wells and the Petri dishes were incubated at 37 °C. After 24–48 h of incubation, the inhibition zones (mm) were recorded.

### Lab-scale production of wheat bread

Different proportions of sourdough were used: 5%, 7% or 10% of the 96-h sourdough with the *two-strain* starter, and 5% or 7% of the sourdough with the *four-strain* starter. Each dough was prepared with 1.5% NaCl, 2% yeast starter, the respective percentage of sourdough and tap water (the amount of water depends on the water absorption of the type of flour). The dough was kneaded in a mixer: slow kneading (1000 r/min) for 4 min and fast kneading (1400 r/min) for 10 min. After that, the dough was rested for about 10 min in order for its elastic properties to be improved. Loaves were formed and were allowed to leaven in forms for about 40–45 min at 30 °C and relative humidity (RH) of 80 ± 5 RH in the production laboratory. Baking was carried out at 225 ± 5 °C for 30 min in a deck oven. Loaves were allowed to cool for 120 min at room temperature. Control bread (bread without sourdough with starter) was baked, cooled and evaluated in parallel.

The baked bread with different amounts (in %) and types of sourdough was evaluated by seven trained judges on the basis of six criteria: aroma, taste, softness of the crumb, colour of the crumb, colour of the crust and volume of the loaf. A scale of 0 (worst quality) to 10 (best quality) was used for each criterion.

### Evaluation of bacterial and mold spoilage of baked bread

The determination of bacterial and mold spoilage of the baked breads was conducted with highly contaminated flours, i.e. containing high concentration of *Bacillus* spores (over 10^2^ cfu/g). Baked breads with 10% or 15% of the 96-h sourdough with the two-strain and the four-strain starters were incubated in non-aseptic conditions in parallel experiments at room temperature and in a thermostat at 37 °C for 72 h for bacterial spoilage and at room temperature and in a thermostat at 30 °C for 96 h for mold spoilage. The appearance of bacterial and mold spoilage was evaluated by 10 trained judges in the production laboratory. For bacterial spoilage, a scale of I–IV was used: I – barely noticeable (pleasant fruity odour); II – weak (distinct change in the odour); III – medium (moisty, sticky crumb, sharp odour); IV – strong (unpleasant odour, brown-yellow crumb). Mold spoilage was evaluated by the appearance of single mold colonies.

### Statistical analysis

All analyses were performed in triplicate and the mean values and standard deviations were determined using MS Office Excel 2007.

## Results and discussion

### Selection of strains

**Figure 1.**
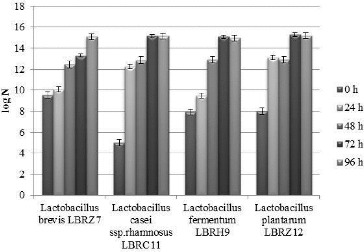
Concentration of viable cells of lactobacilli of sourdough at repeated daily kneading for 96 h.

In a series of experiments, the ability of the four *Lactobacillus* strains to grow in a flour/water mixture was investigated. The initial concentration of LAB in the flour was 10^3^ cfu/g. On the 24th hour, it was below 10^5^ cfu/g, while the concentration of wild yeasts reached over 10^8^ cfu/g, and after the 24th hour, the fermentation of the control sourdough became alcoholic. The initial concentrations of the 24-h single-strain cultural suspensions used for the preparation of the single-strain sourdoughs are given in Table 1. The results of the parallel studies of the single-strain sourdoughs are shown in Table 2 and Figure 1. All four strains of lactobacilli grew well in a flour/water mixture, reaching 10^14^–10^15^ cfu/cm^3^ within 96 h (Figure 1). The TTA values of the resulting sourdough types increased to over 10 °N (Table 2).

**Table 1.** Total titratable acidity and concentration of viable cells of the four 24-h suspensions of the four *Lactobacillus* strains.

Strain	TTA (°T)	*N* (cfu/cm^3^)
*L. brevis* LBRZ7	157.08	5.2 × 10^12^
*L. casei* ssp. *rhamnosus* LBRC11	75.48	8.0 × 10^10^
*L. fermentum* LBRH9	118.93	1.3 × 10^13^
*L. plantarum* LBRZ12	116.69	2.4 × 10^12^

**Table 2.** Changes in the total titratable acidity (TTA (°N)) of single-strain sourdough at repeated daily kneading for 96 h.

Time (h)	0	24	48	72	96
Single-strain sourdough					
*L. brevis* LBRZ7	1.7	5.7	11.2	11.1	10.4
*L. casei* ssp*. rhamnosus* LBRC11	1.4	8.1	10.9	10.4	10.1
*L. fermentum* LBRH9	1.8	5.3	9.8	10.6	10.2
*L. plantarum* LBRZ12	1.7	10.7	10.4	10.1	10.2

By the 24th hour, each of the four sourdoughs possessed specific aroma. The strains *L. fermentum* LBRH9 and *L. brevis* LBRZ7 produced CO_2_ and the volumes of these sourdoughs were visibly greater than the volumes of the other two sourdoughs. At the 48th hour at repeated kneading every 24 h, the four single-strain sourdoughs had a different type and strength of aroma than at the 24th hour, but by the 72nd hour at repeated kneading, the sourdoughs were with identical cheesy aroma, which was retained by the 96th hour.

**Figure 2.**
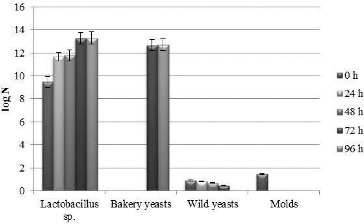
Concentration of viable cells of lactobacilli, molds and yeasts in the four-strain starter sourdough during repeated kneading every 24 h over a period of 96 h.

**Figure 3.**
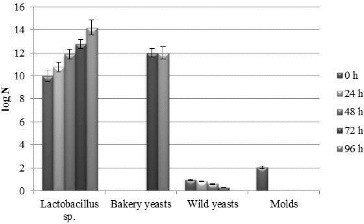
Change in the concentration of viable cells of lactobacilli, molds and yeasts in the two-strain starter sourdough during repeated kneading every 24 h over a period of 96 h.

The four strains were previously demonstrated not to inhibit the growth of the *S. cerevisiae* strain used in the production laboratory and that they inhibit to a different extent some of the most common saprophytes, associated with bread spoilage (*B. subtilis*, *Penicillium* sp., *A. niger* and *Rhizopus* sp.).[42,43] In the present study, a two-strain and a four-strain starter combination were designed. The *Lactobacillus* strains and their ratios in the four-strain combination were selected based on our preliminary experiments (unpublished data) and reference information that *L. brevis* and *L. plantarum* are considered to have the most appropriate profiles of flavour components.[25,44] The ratios and the strains in the two-strain combination were selected arbitrarily. The accumulation of biomass and the change in the acidity in the two sourdoughs with the two starters are given in Figure 2, Figure 3 and Table 3. The four *Lactobacillus* strains in the sourdough with the four-strain starter grew with accumulation of high concentrations of viable cells (over 10^10^ cfu/g) of lactobacilli (Figure 2) and increase in the titratable acidity to 17.3 °N (Table 3). In the sourdough with the two-strain starter, the number of viable cells reached 10^14^ cfu/g (Figure 3), and the titratable acidity was 1 °N lower than that of the sourdough with the four-strain starter (Table 3). In both sourdoughs, inhibition of the growth of wild yeasts and molds but not of that of the baker's yeasts was observed (Figures 2 and  3), which is partially due to the metabolites formed by LAB in the composition of the starters. This ability is particularly important in the fermentation of the dough for the production of bread and for the repeated kneading of the sourdoughs over a period of six months.

**Table 3.** Changes in the total titratable acidity (TTA (°N)) in wheat sourdough with starter cultures that are kneaded repeatedly every 24 h over a period of 96 h.

Time (h)	0	24	48	72	96
Wheat sourdough					
Four-strain starter	2.5	9.0	8.4	12.7	17.3
Two-strain starter	2.9	8.8	10.4	15.0	16.4

The antimicrobial activity of the two 96-h sourdoughs with the four-strain starter and the two-strain starter against some of the most common saprophytes associated with bread spoilage was determined by the agar diffusion method. The two sourdoughs were shown to inhibit the growth of *B. subtilis*, *A. niger*, *Penicillium* sp. and *Rhizopus* sp., but they did not affect the growth of *S. cerevisiae* (Table 4). The observed antimicrobial activity of the sourdough with the ‘four-strain’ starter, containing *L. plantarum* LBRZ12, was partially due to the fact that *L. plantarum* LBRZ12 can inhibit the growth of rope-forming *B. subtilis* strains through bacteriocin and acidity production.[12,23,32,44]

**Table 4.** Antimicrobial activity of the two 96-h sourdoughs against *Bacillus subtilis*, *Aspergillus niger*, *Saccharomyces cerevisiae*, *Penicillium* sp. and *Rhizopus* sp. *d* (mm) well = 6 mm.

Sourdough with starter	Four-strain starter	Two-strain starter
Saprophyte		
*Bacillus subtilis* 3.5 × 10^5^ cfu/cm^3^	15.2	15.0
*Aspergillus niger* 6.4 × 10^4^ cfu/cm^3^	10.5	10.0
*Saccharomyces cerevisiae* 8 × 10^4^ cfu/cm^3^	–	–
*Penicillium sp*. 8 × 10^4^ cfu/cm^3^	15.3	15.0
*Rhizopus sp*. 3.2 × 10^4^ cfu/cm^3^	10.5	10.0

It was determined that after 48–72 h of incubation with repeated kneading every 24 h, the two types of sourdough reached the consistency typical for sourdough and were characterized by pleasant lactic acid aroma, more pronounced in the sourdough with the two-strain starter.

### Lab-scale production of wheat bread

**Figure 4.**
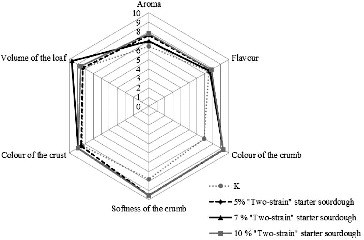
Evaluation of the variants of bread with sourdough with the two-strain starter.

**Figure 5.**
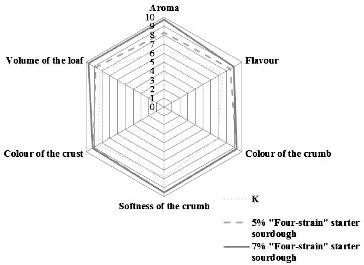
Evaluation of the variants of bread with sourdough with the four-strain starter.

Bread was baked with 5%, 7% or 10% of the sourdough with the two-strain starter and with 5% or 7% of the sourdough with the four-strain starter. The results of their evaluation according to six criteria: aroma, taste, softness of the crumb, colour of the crumb, crust colour and volume of the loaf, are given in Figures 4 and 5.

The presence of *L. plantarum* strains in the starter was previously observed to generally enhance the fermentation activities in the dough.[45,46] The sourdoughs with the starters were stronger and more elastic, the volume of the pieces of bread before and after baking was greater than that of bread without sourdough, the taste and the aroma of the final bread were improved. The wheat bread with the starter sourdough has softer and lighter crumb, with a pleasant and characteristic lactic acid aroma.

Bread with the best characteristics was obtained using the sourdough with the four-strain starter in a quantitative ratio of 7% and, among the variants of bread with sourdough with the two-strain starter, the most successful option was again the bread with 7% sourdough (Figures 4 and 5).

### Bacterial and mold spoilage

Our experiments demonstrated that bacterial spoilage due to the growth of *Bacillus* representatives occurred earlier in the control loaf incubated at 37 °C than in the one incubated at room temperature. The results showed that in loaves baked with 10% of the sourdough with the two-strain starter or the four-strain starter, bacterial spoilage became noticeable after the 48th hour both at room temperature and at 37 °C, except for the loaf with 10% of the sourdough with the four-strain starter at 37 °C, where bacterial spoilage was delayed by an additional 24 h. In loaves with 15% of the sourdough with the two-strain starter or the four-strain starter, bacterial spoilage was not noticeable even at the 72nd hour after taking the loaves out of the oven at room temperature and at 37 °C (Table 5).

**Table 5.** Bacterial bread spoilage by *Bacillus* sp. during incubation of baked breads for 24–72 h at room temperature (RT) or 37 °C.

		24 h		48 h		72 h	
Bread	*T* (°C)	Degree of spoilage	Odour	Degree of spoilage	Odour	Degree of spoilage	Odour
Control (without sourdough)	RT	–	No	–	No	I	Yes
	37	–	No	I	Yes	II	Yes
Two-strain starter sourdough 10%	RT	–	No	–	No	I	Yes
	37	–	No	–	No	I	Yes
Two-strain starter sourdough 15%	RT	–	No	–	No	I	Yes
	37	–	No	–	No	I	Yes
Four-strain starter sourdough 10%	RT	–	No	–	No	–	No
	37	–	No	–	No	I	Yes
Four-strain starter sourdough 15%	RT	–	No	–	No	–	No
	37	–	No	–	No	–	No

Mold spoilage was observed in the control loaves and in the loaves with 10% of the sourdough with the two-strain starter after the 72nd hour after baking, while in all other variants mold spoilage did not become noticeable by the 96th hour (Table 6).

**Table 6.** Mold bread spoilage during incubation of the baked breads at 30 °C and at room temperature (RT).

		24 h		48 h		72 h		96 h	
Bread	*T* (°C)	Degree of spoilage	Odour	Degree of spoilage	Odour	Degree of spoilage	Odour	Degree of spoilage	Odour
Control (without sourdough)	RT	–	No	–	No	–	No	I	Yes
	30	–	No	–	No	–	No	I	Yes
Two-strain starter sourdough 10%	RT	–	No	–	No	–	No	I	Yes
	30	–	No	–	No	–	No	I	Yes
Two-strain starter sourdough 15%	RT	–	No	–	No	–	No	–	No
	30	–	No	–	No	–	No	–	No
Four-strain starter sourdough 10%	RT	–	No	–	No	–	No	–	No
	30	–	No	–	No	–	No	–	No
Four-strain starter sourdough 15%	RT	–	No	–	No	–	No	–	No
	30	–	No	–	No	–	No	–	No

These results demonstrate that the loaves with the sourdough with the four-strain starter were characterized by delayed onset for bacterial and mold spoilage compared to those with the sourdough with the two-strain starter when the two types of sourdough were administered in a ratio of 10%. Moreover, when 15% sourdough was added in the process of bread making, there was no bacterial or mold spoilage even 72 or 96 h after baking, respectively, both at room temperature and at 37 or 30 °C. This suggests that the combination of four strains in a starter for sourdough led to the production of a better quantity and quality combination of metabolites in the sourdough, which affected positively the storage time of the baked bread (Tables 5 and 6).

The incorporation of 15% or more sourdough with the developed four-strain starter results in bread with elongated shelf life. This is in agreement with other results that the incorporation of 15% or more sourdough in the bread-making process inhibits the growth of bacterial and mold spores and ensures long shelf life of the baked bread.[47] While the increase in acidification may be necessary for optimal swelling and baking of bread, for the control of enzymatic activities, elasticity and suitability of the crumb, and for prolonging the shelf life,[32,48] excessive acidification has, in contrast, a deleterious effect on some rheological parameters [45,49]. Although, in our study, sourdough was incorporated in a ratio of 15% in non-sterile (non-aseptic) conditions in contrast to the experiments of Mentes et al. [47], which were conducted in aseptic conditions, our results also demonstrated prevention of bacterial and mold spoilage during incubation of the baked bread at room temperature (25–30 °C).

## Conclusions

Four *Lactobacillus* strains were included in starters for sourdough for the production of wheat bread. Two multi-strain starters for sourdough were developed: a two-strain combination (*L. casei* ssp. *rhamnosus* LBRC11 and *L. brevis* LBRZ7 in a 3:7 ratio) and a four-strain combination (*L. plantarum* LBRZ12, *L. casei* ssp. *rhamnosus* LBRC11, *L. brevis* LBRZ7 and *L. fermentum* LBRH9 in a 2:1:1:1 ratio). When the two types of sourdough were included in the bread-making process in a ratio of 7%, the technological and organoleptic (taste and aroma) characteristics of the final wheat bread were improved without the addition of preservatives. The breads baked with sourdough had a softer crumb lighter in colour, pleasant lactic acid odour and darker colour of the crust in comparison to the control ones without a starter. The inclusion of the two types of sourdough in the bread-making process in a ratio of 15% prevented bacterial and mold spoilage. The two starters differentially affected the quality and shelf life of the baked bread. The obtained results are a fundamental stage in the selection of microbial flora to be employed in a variety of baked products to satisfy the complex demands of the modern food industry.
